# Fair and Safe Eligibility Criteria for Women's Sport: The Proposed Testing Regime Is Not Justified, Ethical, or Viable

**DOI:** 10.1111/sms.14753

**Published:** 2024-11-04

**Authors:** Alun G. Williams, Shane M. Heffernan, Adam J. Herbert, Blair R. Hamilton, Francisco J. Sánchez, Sasha Gollish, Adam Rutherford, Hugh E. Montgomery, Mike McNamee, Silvia Camporesi, Jonathan Ospina‐Betancurt, Niall Fife, Luke Cox, Richard I. G. Holt, Yannis P. Pitsiladis, Fernanda R. Malinsky, Fergus Guppy, Madeleine Pape, Eric Vilain, Roger Pielke, N. Tim Cable, Sarah Chantler, Stuart M. Phillips, Georgina K. Stebbings

**Affiliations:** ^1^ Manchester Metropolitan Institute of Sport Manchester Metropolitan University Manchester UK; ^2^ Institute of Sport, Exercise and Health University College London London UK; ^3^ Applied Sports, Technology, Exercise and Medicine Research Centre (A‐STEM), Faculty of Science and Engineering Swansea University Swansea UK; ^4^ Centre for Life and Sport Sciences Birmingham City University Birmingham UK; ^5^ The Gender Identity Clinic Tavistock and Portman NHS Foundation Trust London UK; ^6^ School of Counseling and Counseling Psychology Arizona State University Tempe Arizona USA; ^7^ Faculty of Kinesiology and Physical Education, Mental Health and Physical Activity Research Centre University of Toronto Toronto Ontario Canada; ^8^ Department of Genetics, Evolution and Environment University College London London UK; ^9^ Division of Medicine University College London London UK; ^10^ Department of Movement Sciences KU Leuven Leuven Belgium; ^11^ Department of Public Health and Primary Care, Centre for Biomedical Ethics and Law KU Leuven Leuven Belgium; ^12^ Departamento Didáctica de la Expresión Corporal, Facultad de Educación y Trabajo Social Universidad de Valladolid (UVA) Valladolid Spain; ^13^ Human Development and Health, Faculty of Medicine University of Southampton Southampton UK; ^14^ Southampton National Institute for Health Research Biomedical Research Centre University Hospital Southampton NHS Foundation Trust Southampton UK; ^15^ Department of Sport, Physical Education and Health Hong Kong Baptist University Hong Kong China; ^16^ International Federation of Sports Medicine (FIMS) Lausanne Switzerland; ^17^ School of Energy, Geosciences, Infrastructure and Society, Institute of Life and Earth Sciences Heriot Watt University Edinburgh UK; ^18^ Institute of Social Sciences University of Lausanne Lausanne Switzerland; ^19^ Institute for Clinical and Translational Science University of California Irvine Irvine California USA; ^20^ University of Colorado Boulder Boulder Colorado USA; ^21^ Carnegie School of Sport Leeds Beckett University Leeds UK; ^22^ Department of Kinesiology McMaster University Hamilton Ontario Canada

In an invited editorial, Tucker et al. [1] addressed the eligibility controversy regarding the Paris 2024 Olympic boxing competition. They cited Lundberg et al. [2] concerning the in/eligibility of transgender women for the female sports category and identified performance differences between males and females alongside studies involving testosterone suppression. Several authors of the present letter also co‐authored Lundberg et al. [2] and stand by that paper, but declined co‐authorship of Tucker et al.'s editorial [1] and present here, with additional collaborators, challenges to that editorial.

First, the editorial [1] did not acknowledge the absence of high‐quality scientific data regarding sport performance advantage of athletes with XY differences of sex development (DSDs). Furthermore, individuals with DSDs possess one or more of numerous rare genetic mutations [3], causing wide variability in pubertal physical development relevant to sport performance between and within different DSDs. Consequently, no primary evidence base currently exists demonstrating athletic performance advantage to justify testing and regulation of an entire population of competitors.

Second, despite that lacuna, Tucker et al. [1] propose mandatory genetic testing in women's sports worldwide in “early”, sub‐elite competition. Figure 2 in Lundberg et al. [2] includes high‐performing teenage athletes. If consistent with their thesis, “early” must therefore include minors. Mandatory genetic testing was abandoned in 1999 due to concerns about validity, financial cost, and practicality, as well as trauma and stigmatization for many athletes [4]. All these concerns remain and are amplified by the vastly increased number of younger athletes tested under Tucker et al.'s [1] proposal. The editorial [1] gives the impression that such tests are straightforward, “individual consent, confidentiality, and dignity… simple cheek swab… standard medical care”, but these assurances ignore the enormous problems such a testing regime would generate.

Individual voluntary consent for genetic testing is a core principle [5]. Under Tucker et al.'s [1] proposed mandatory genetic testing for sport eligibility rather than healthcare, young athletes would not be presented with a genuine choice. Consent is only a coercive offer: comply with the test or never participate in competitive women's or girls' sport, even at sub‐elite level. Furthermore, ethically responsible genetic counseling ensures people understand the potential consequences of receiving genetic test results before consenting [6] and provides comprehensive professional follow‐up [7]. Who would fund or produce this worldwide army of counseling expertise? For those undergoing follow‐up clinical examination and genome sequencing (only providing a genetic diagnosis in ~50% of cases [8]), how would the devastation of young athletes' personal identity and self‐esteem, and the alarm caused to their families [9], be managed? The resultant duty of care of these athletes will fall to the sport federations mandating such assessments, without any realistic prospect of being fulfilled.

What, precisely, does follow‐up clinical examination involve? Tucker et al. [1] say only “standard medical care”. They do not state transparently that it begins with clinical examination to assess clitoromegaly, symmetry of external genital structures, presence/absence of breast development, extent of sexual hair, involves palpation of genitalia, and so forth [9]. Indeed, as part of a “Level 1 Assessment”, World Athletics regulations [10] require such clinical examination by gynecologists or physicians following current guidance [9]. The concerns outlined above also apply to the World Athletics regulations, but even these affect only a small proportion of the athletes that would have to undergo such invasive and potentially humiliating examination under the editorial's [1] proposal.

We agree with the editorial's [1] criticism of the current approach in some sports, which “invites targeted testing based on allegation, suspicion, subjective assessment, and bias” and broad discussion is required to develop more appropriate regulations. However, the proposed mandatory testing of all young women and girls in sport is not justified by scientific evidence, has limited ethical defensibility, and is not an operationally viable proposition.

## Conflicts of Interest

The authors would like to make a joint conflict of interest statement in which we declare the following: Several authors have received payment to provide expert testimony related to this topic and/or served as pro bono expert witnesses in relevant proceedings before the Court of Arbitration for Sport. Several authors have received payment for work with sports organisations. Several authors have received travel and accommodation expenses for speaking engagements related to this topic. Several authors have spoken in the mainstream media on this topic. Several authors have received research funding from relevant organisations including the International Olympic Committee, the World Anti‐Doping Agency and the US Anti‐Doping Agency.

## Data Availability

Data sharing is not applicable to this article as no new data were created or analyzed in this study.
